# Modified Sijunzi granule decreases post-weaning diarrhea in Rex rabbits *via* promoting intestinal development

**DOI:** 10.3389/fvets.2022.972326

**Published:** 2022-11-07

**Authors:** Dongbo Li, Yueli Wang, Ning Liu, Shiqi Chen, Hanzhong Liu, Ping Wang, Zhiju Yu, Gang Shu, Juchun Lin, Wei Zhang, Guangneng Peng, Ling Zhao, Huaqiao Tang, Kai Zhang, Bin Wen, Hualin Fu

**Affiliations:** ^1^Department of Pharmacy, College of Veterinary Medicine, Sichuan Agricultural University, Chengdu, China; ^2^Sichuan Academy of Grassland Sciences, Chengdu, China

**Keywords:** modified Sijunzi granule, post-weaning diarrhea, SIgA, intestinal mucosal structure, tight junction protein, glucose transporter

## Abstract

Traditional Chinese medicine (TCM) formulas can be adjusted on the basis of TCM basic theory to achieve the best curative effect, especially for diseases with complex pathogenesis, such as post-weaning diarrhea (PWD). Shugan Jianwei Sijunzi decoction (SJ-SJZD) can be recognized as modified Sijunzi Decoction (SJZD) supplemented with *Astragalus mongholicus Bunge, Bupleurum chinense DC, Citrus* × *aurantium L., and Crataegus pinnatifida Bunge (fruit)* in a fixed dosage ratio. The inactive ingredients were subsequently added to make granule, which was Shugan Jianwei Sijunzi granule (SJ-SJZG). Previous studies have confirmed the antagonism of SJ-SJZG to PWD. However, the mechanism of SJ-SJZG protective effects on small intestine in weaned Rex rabbits remained unclear. Animals were randomly divided into negative control (NC), low dose (LD), medium dose (MD), high dose (HD), and positive control (PC). SJ-SJZG significantly increased the intestinal length and the jejunum villi length. The SIgA level was statistically increased in duodenum and jejunum with the ELISA. Immunohistochemical detection showed that SIgA protein expression was also increased significantly in jejunum. Meanwhile, the relative expression of Zo1 in duodenum and jejunum of SJ-SJZG group increased significantly. SJ-SJZG significantly increased the relative expression of occludin in duodenum and jejunum as well. Moreover, real-time PCR results showed a significant increase in GLUT2 and SGLT1 relative expression in ileum. SJ-SJZG could also obviously enhance the expression of GLUT2 in jejunum and the expression of SGLT1 in duodenum. In conclusion, SJ-SJZG had been proven to be effective in promoting the development of small intestine and improving the immunity of small intestine. Moreover, SJ-SJZG could ensure the integrity of mucosal barrier and increase the ability of intestine to absorb glucose in small intestine.

## Introduction

Rex rabbit, as a great economic return animal, has excellent fur and grows rapidly (1). However, Rex rabbits are prone to diarrhea and even death after weaning, which is called post-weaning diarrhea (PWD), reducing their breeding and affecting economic benefits (2). It is due to the incomplete intestinal development of weaned Rex rabbits. When Rex rabbits were weaned, due to the lack of breast milk antigen, the intestinal immunity decreased rapidly (3). Moreover, the tight junction between the small intestinal epithelial cells of weaned rabbits was not as close as adult rabbits. The intestinal wall was very thin and the intestinal permeability is high, which lead to the destruction and invasion of intestinal mucosa by incomplete digestion products and toxins (3). The villi and epithelial cells of the small intestine of weaned Rex rabbits were further damaged, which exacerbated the symptoms of diarrhea and led to death.

Sijunzi Decoction (SJZD), as a traditional Chinese medicine (TCM) prescription, was well-known for treating disorders of digestive function manifested by poor appetite, indigestion, and diarrhea and mostly used to treat spleen (Qi) deficiency. In TCM theory, the Qi deficiency will lead to a lack of biochemical source and blood, poor food digestion and absorption, and decline of body immunity, leading to functional diarrhea and other gastrointestinal diseases, which are also the symptoms of stress-induced diarrhea in weaned rabbits. Modern research had confirmed the mechanism of SJZD in the treatment of spleen deficiency (4). Recent studies had also shown that SJZD could regulate the expression of intestinal immune factor genes and proteins (5). Besides that, researchers revealed that SJZD may promote intestinal epithelial restitution after wounding (6). Hence, SJZD had great potential in the treatment of diarrhea symptoms of weaned Rex rabbits. Accumulated studies had shown that SJZD can enhance intestinal immunity, promote intestinal development, and restore intestinal barrier function (7, 8).

Moreover, SJZD has also been modified to own wider pharmacological actions based on the original major formula and TCM theory to fit different clinical demands. All modified SJZD used SJZD as a major formula and combined it with the other TCMs that possess synergistic or additive activity to promote the function of the SJZD. For example, supplementary SJZD can improve the digestion and absorption of small intestine and promote the expression of small intestinal growth factor (9); Qilan SJZD can increase the mRNA expression level of small intestinal cytokines and improve the level of cellular immunity (10); modified SJZD can enhance intestinal absorption of nutrients (11).

In the present study, we added *Astragalus mongholicus Bunge, Bupleurum chinense DC, Citrus* × *aurantium L.*, and *Crataegus pinnatifida Bunge (fruit)* to the formula of the original SJZD, *Codonopsis pilosula (Franch.) Nannf*, *Atractylodes Lancea (Thunb.) DC, Wolfiporia cocos, Glycyrrhiza uralensis Fisch. ex DC* (Preparata), as a modified SJZD, which is called Shugan Jianwei Sijunzi Decoction. Shugan Jianwei Sijunzi granule (SJ-SJZG) was used as a granulated extract obtained by adding inactive ingredients to the hot water extract of a mixture of the above eight crude herbs.

Previous studies had shown that the protective effect of SJ-SJZG on PWD in Rex rabbits was confirmed by growth performance, diarrhea frequency, and mortality, but the mechanisms were unclear (12, 13). To further explore the development effect of SJ-SJZG on weaned Rex rabbits, especially on intestinal development, we investigated the effects of SJ-SJZG on the morphological structure of small intestine, mucosal immunity, and the gene expression of tight junction protein and glucose transporter in weaned Rex rabbits, aiming to provide a theoretical basis for the research of SJ-SJZG and its application in production.

## Materials and methods

### Chemicals and reagents

Glutamine was purchased from Huana Chemical Co., Ltd. (China). HE staining (hematoxylin and eosin) was purchased from Thermo Fisher Co., Ltd. (America). SIgA ELISA kits were purchased from Nanjing Jiancheng Bioengineering Institute. SIgA rabbit polyclonal antibody was obtained from Youning Weisheng Technology Co., Ltd. (Shanghai). FastKing RT kits were purchased from Tiangen Biotech Co., Ltd. (Beijing). The other reagents were purchased from Chengdu Chron Chemicals Co., Ltd. (China).

### Animals

**Figure 1:**
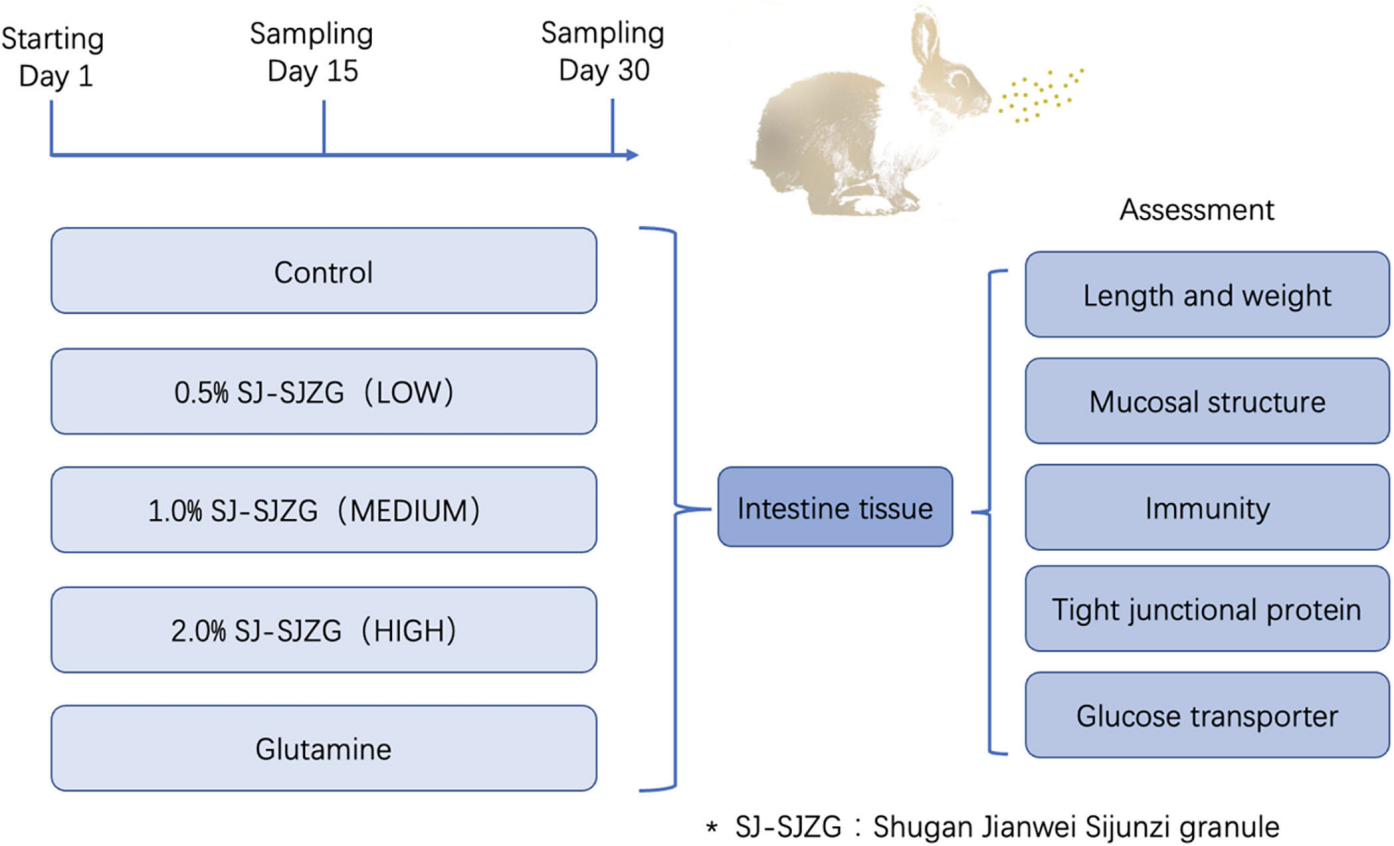
Experimental design of this study.

A total of 160 healthy Sichuan White Rex rabbits, half male and half female, aged 45 days and weighing about 850 g were provided by Rex Rabbit Research Institute of Sichuan Academy of grassland sciences. The animals were provided with basal diet and tap water at liberty and maintained in cages under controlled conditions (23 ± 2°C, 12-h light/dark cycle). All experiments and procedures were carried out according to the Regulations of Experimental Animal Administration issued by the State Committee of Science and Technology of China. The composition and nutrient levels of the basal diet are listed in Table 1. After 7 days of adaptive feeding, animals were randomly divided into five groups, 32 in each group, including negative control (NC), low dose (LD), medium dose (MD), high dose (HD), and positive control (PC). The NC group was fed with the basic diet above. SJ-SJZG (0.5, 1, and 2%) was administrated in LD, MD, and HD groups, respectively, as a dietary supplement for 30 consecutive days. Similarly, for PC group, SJ-SJZG was replaced by 0.8% glutamine. In each group, six rabbits were randomly selected, weighted, and sacrificed on the 15th day after treatment, respectively. The intestinal length and relative weight were measured after washing by physiological saline. Then, two samples of duodenum, jejunum, and ileum were collected, respectively, fixed with 4% paraformaldehyde, or preserved in liquid nitrogen. On the 30th day of the experiment, another six rabbits in each group were killed at random, and the sampling was as above. All animal procedures were in accordance with the national standard, Laboratory Animal-Requirements of Environment and Housing Facilities (GB14925-2001), the Sichuan Agricultural University Institutional Animal Care and Use Committee under permit number CSQ-2018203003. The detailed experimental design is shown in Figure 1.

**Table 1:** The composition and nutrient levels of basal diet (air-dry basis) %.

**Items**	**Content**	**Nutrient levels**	**Content**
Ingredients		Digestible energy (DE, MJ/kg)	10.21
Corn	20.0	Crude protein (CP)	16.00
Bran	22.8	Crude fiber (CF)	14.29
Peanut vine	40.1	EE	3.02
Soyben meal	14.6	Met+Cys	0.46
NaCl	0.5	Lys	0.56
CaHPO_4_	1.0	Ca	0.58
Premix	1.0	P	0.62
Total	100	I (mg/kg)	0.22

The premix provided following per kilogram of diet: Lys 1.5 g; VA 8,000 IU; VD 3 1,000 IU; VE 50 mg; Fe 100 mg; Cu 50 mg; Mg 150 mg; Zn 50 mg; Mn 30 mg; Se 0.1 mg.

Nutrition levels were calculated values.

### Preparation of SJ-SJZG

SJ-SJZG was prepared according to the method of the previous studies but a little improvement in our research group (12). In brief, eight herbs, Codonopsis pilosula (Franch.) Nannf, Atractylodes Lancea (Thunb.) DC, Wolfiporia cocos, Glycyrrhiza uralensis Fisch. ex DC (Preparata), Astragalus mongholicus Bunge, Bupleurum chinense DC, Citrus × aurantium L., and Crataegus pinnatifida Bunge (fruit) with a dosage ratio of 9:9:9:6:10:6:6:6, were mixed and wetted with distilled water (1:8, w/v) for 2 h. Then, we boiled the herbs for 3 h, filtered it with multi-layer gauze, and collected the filtrate. The herbs were added with water (1:6, w/v) and boiled for 3 h twice, filtered with multi-layer gauze similarly. All the filtrates were mixed and concentrated for granulation. The extracts were granulated by adding inactive ingredients and wetting agents. The concentration of SJ-SJZG was regarded as each gram of granules contained one gram of raw plant drugs. All the above herbs were purchased from Sichuan C&Y Traditional Chinese Medicine CO., LTD.

### High-performance liquid chromatography (HPLC)

**Figure 2:**
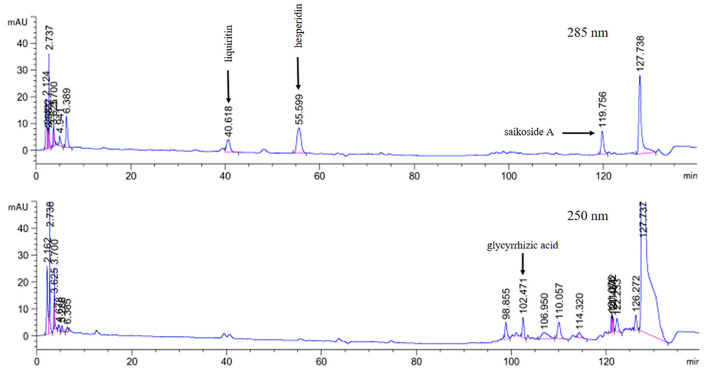
Qualitative and quantitative analysis of SJ-SJZG. The retention times of liquriritin, hesperidin, and saikoside A at 285 nm were 40.62, 55.60, and 119.76 min respectively; the retention times for glycyrrhizic acid at 250 nm was 102.47 min.

The SJ-SJZG was analyzed using high-performance liquid chromatography (HPLC) with Corona ultra-detection (CAD) in Agilent HPLC system. In brief, 5 g SJ-SJZG was dissolved in 10 mL 70% methanol solution and diluted 10 times. The solution was subjected to centrifugation at 1,500 rpm and 15 min, and the supernatant was filtered through a 0.22-μm filter. Subsequently, 20 μL supernatant was injected into HPLC system for analysis. The chromatographic conditions were as follows: Reverse-phase column (Inertsil ODS-3, 5 μm, 4.6 mm× 250 mm I.D.) connected with a guard column (C18, 5 μm, 4.6 mm× 10 mm I.D.). The elution flow rate was 1.0 mL/min with a mobile phase gradient of A-B (A: H_2_O/H_3_PO_4_ = 1,000 mL/2 mL; B: CH_3_CN), which was varied as follows: 0min, 90% A, 10% B; 9~29 min, 90~85% A, 10~15% B; 29~37 min, 85~82% A, 15~18% B; 37~46 min, 82% A, 18% B; 46~51 min, 82~80%A, 18~20% B; 51~72 min, 80~72% A, 20~28% B; 72~82 min, 72% A, 28% B; 82~87 min, 72~70% A, 28~30% B; 87~92 min, 70~67% A, 30~33% B; 92~99 min, 67~50% A, 33~50% B; 99~110 min, 50% A, 50% B; 110~115 min, 50~47% A, 50~53% B; 115~118 min, 47~30% A, 53~70% B; 118~130 min, 30% A, 70% B; 0min, 90% A, 10% B; 130~135 min, 30~90% A, 70~10% B; 135~140 min, 90% A, 10% B. The injection volume was 20 μl, and the UV detection wavelength was set at 285 nm for saikoside A, liquiritin, and hesperidin and 250 nm for glycyrrhizic acid. In the quantitative analysis of active components of SJ-SJZG, the concentrations of saikoside A, liquiritin, hesperidin, and glycyrrhizic acid were 0.645, 0.652, 1.161, and 0.398 mg/g in SJ-SJZG, respectively (Figure 2).

### Histopathological examination (H&E) staining

The small intestine tissues fixed with 4% paraformaldehyde were washed with running water for 30 min. Subsequently, they were put into the pathological embedding plastic basket for dehydration to transparent with gradient alcohol and finally embedded in paraffin. The tissues were sliced into 5 μm thick slices by the slicer (Ultra-Thin Semiautomatic Microtome, RM2235, from Leica, Germany), flattened in warm water, fished on the slide, and baked at 60°C for 2h. Then, the sections were placed in xylene to remove the paraffin and stained with hematoxylin and eosin. Finally, the tissues were dehydrated with gradient alcohol, put in xylene making them transparent, and sealed with resin glue.

### Histomorphology

The slides were observed under the CX22 microscope (Leica, Germany), and the whole tissue slice was browsed completely by DM 1000 Leica microscopic imaging system. Image pro Plus 6.0 (Media Cybernetics, America) was used to measure the length of villi and the depth of crypt, and at least 10 intact villi and corresponding crypts in intestinal tissue were measured. Their ratio was calculated as follows (Equation 1).


(1) $$\begin{array}{l} R_{\left(\frac{V}{C}\right)}=\frac{L_{villi}}{D_{crypt}} \end{array}$$

Where L_villi_ and D_crypts_ stand for the length of villi and the depth of crypt, respectively.

### Immunohistochemical staining

The abundance of SIgA was assessed for paraffin-embedded slides after the sections were dewaxed. Endogen biotin and non-specific signals were blocked with appropriated reagents. Antigen retrieval was carried out in a microwave oven (two cycles for 5 min each at 780 W, in citrate buffer, pH 6.0, twice washed in PBS for 5 min each). Then, the treated slides were overnight incubated with primary antibodies at −4°C in a humidified chamber, washed in PBS, and visualized by biotinylated secondary antibodies followed by staining by DAB kit for 2 min and washed in distilled water, and finally counterstained with hematoxylin, dehydrated, transparentized, and sealed. At least 10 fields of view from each sample by BA400 Digital (Motic China Group CO., LTD.) were analyzed with the imaging system for each protein of interest. All tissues were observed under 100 times of each slice, and then, three visual fields were selected to collect 400 times of microscopic images, respectively. The integrated optical density (IOD) and area of all collected images are measured by Image pro Plus 6.0, and the mean density of each image is calculated. The average optical density of three images is used to calculate the average, and the average optical density of each sample is obtained.

### ELISA

The value of SIgA on the intestinal mucosa was assayed by ELISA kits according to the manufacturer's instructions. The absorbance value of the sample was determined by Varioskan Flash (Thermo Scientific). The data were processed by ELISA scale. The logistic curve (four-parameter) model was used to fit the standard curve between the concentration and the OD value of the standard, and the regression equation is obtained. Then, the OD value of each well sample was brought into the regression equation to calculate the SIgA level.

### Tissue RNA extraction and qRT-PCR assay

Total RNA was isolated from samples of jejunum, ileum, and colon with TRIzol regent (Tiangen Biotech, Beijing) and then treated with DNase I (Tiangen Biotech, Beijing) according to the manufacturer's instructions. Subsequently, we tested the integrity, purity, and concentration of RNA. Fastking cDNA first-strand synthesis Kit, Super real premix plus, 2 × Taq PCR mastermix, and 5 × RNA loading buffer were purchased from Beijing Tiangen Biochemical Technology Co., Ltd. The PCR reaction process was as follows: 95°C for 2 min, followed by amplification in 40 cycles of 95°C for 5 s, 15 s at 60°C, and 20 s at 72°C, and then 65°C and 95°C for 5 s, using the C1000 Touch^TM^ Thermal Cycler Real-Time System (Bio-Rad). Referring to the data of the National Center for Biotechnology Information (NCBI) database, fluorescent quantitative-specific primers were designed by primer 5.0 software. All primers are synthesized by Thermo Fisher Technology Co., Ltd. and are presented in Table 2. GAPDH was used as an internal control to normalize the expression of target gene transcripts.

**Table 2:** Primer sequences for qPCR.

**Gene**	**Geneback**		**Primer sequences (5′ to 3′)**	**bp**	**Temperature°C**
Claudin1	DQ_993356	F	GGAAGATGATGAGGAGCAA	77	59°C
		R	AGCCCAGCCAGTGAAAA		
Occludin	XM_008262318	F	CTTGCCTGGGACAGAACCTA	121	59°C
		R	AGCCATAACCGTAGCCGTAA		
Zo1	XM_008269782	F	GACTGATGCGAAGACGTTGA	117	59°C
		R	GCAGAATGGATGCTGTCAGA		
SGLT1	EU_414633	F	TGTTCCGCAGGGACACTAA	75	59°C
		R	GGGATCAGGACGTAAAGAGG		
GLUT2	XM_01734	F	AGGCACTGTCCACCACC	161	59°C
		R	GTCTCCAAGCCACCCAC		
GAPDH	NM_001082253	F	TGCCACCCACTCCTCTA	163	59°C
		R	AGTAAGAGCCCTCAAACCACCGG		

### Statistical analysis

The one-way analysis of variance (ANOVA) was used to analyze experimental data by SPSS 25.0 software. All values were presented as the mean ± SD, and *P* < 0.05 was considered statistically significant.

## Results

### Growth-promoting effects of SJ-SJZG on small intestine

The length and relative weight of small intestine, reflecting development degree and functional strength, were measured in the two periods, Day 15 and Day 30. The results indicated that SJ-SJZG could increase the small intestinal length of weaned Rex rabbits. The HD group significantly increased the small intestinal length of weaned Rex rabbits. Compared with NC group, it increased by 12.74% at Day 15 and 12.96% at Day 30, respectively, and the same trend was observed in PC group. The relative weight of small intestine in SJ-SJZG groups also increased. However, there was no significant difference between SJ-SJZG groups and NC group due to the rapid growth of Rex rabbits. All the above results are shown in Table 3.

**Table 3:** Effects of SJ-SJZG on intestinal length and relative weight in weaned Rex rabbits.

**Time**	**Item**	**Group**				
		**NC**	**LD**	**MD**	**HD**	**PC**
Day 15	Intestinal length(cm)	273.67 ± 16.42^a^	274.33 ± 36.87^ab^	303.07 ± 24.96^ab^	308.53 ± 33.03^b^	305.00 ± 28.81^ab^
Day 30		282.80 ± 27.57^a^	289.40 ± 29.48^ab^	306.53 ± 36.94^ab^	319.47 ± 25.92^b^	319.00 ± 17.70^b^
Day 15	Relative weight(g/kg)	29.87 ± 5.30	31.90 ± 4.84	32.33 ± 2.35	33.21 ± 4.85	33.70 ± 4.02
Day 30		29.47 ± 1.19	31.63 ± 3.54	32.44 ± 3.11	31.28 ± 1.61	31.53 ± 4.75

Data are expressed as mean ± SD, (n = 6). In the same row, values with no letter or the same letter superscripts means no significant difference (P > 0.05), while with different small letter means no significant difference (P < 0.05). The same as below.

### Effects of SJ-SJZG on intestinal mucosal structure

**Figure 3:**
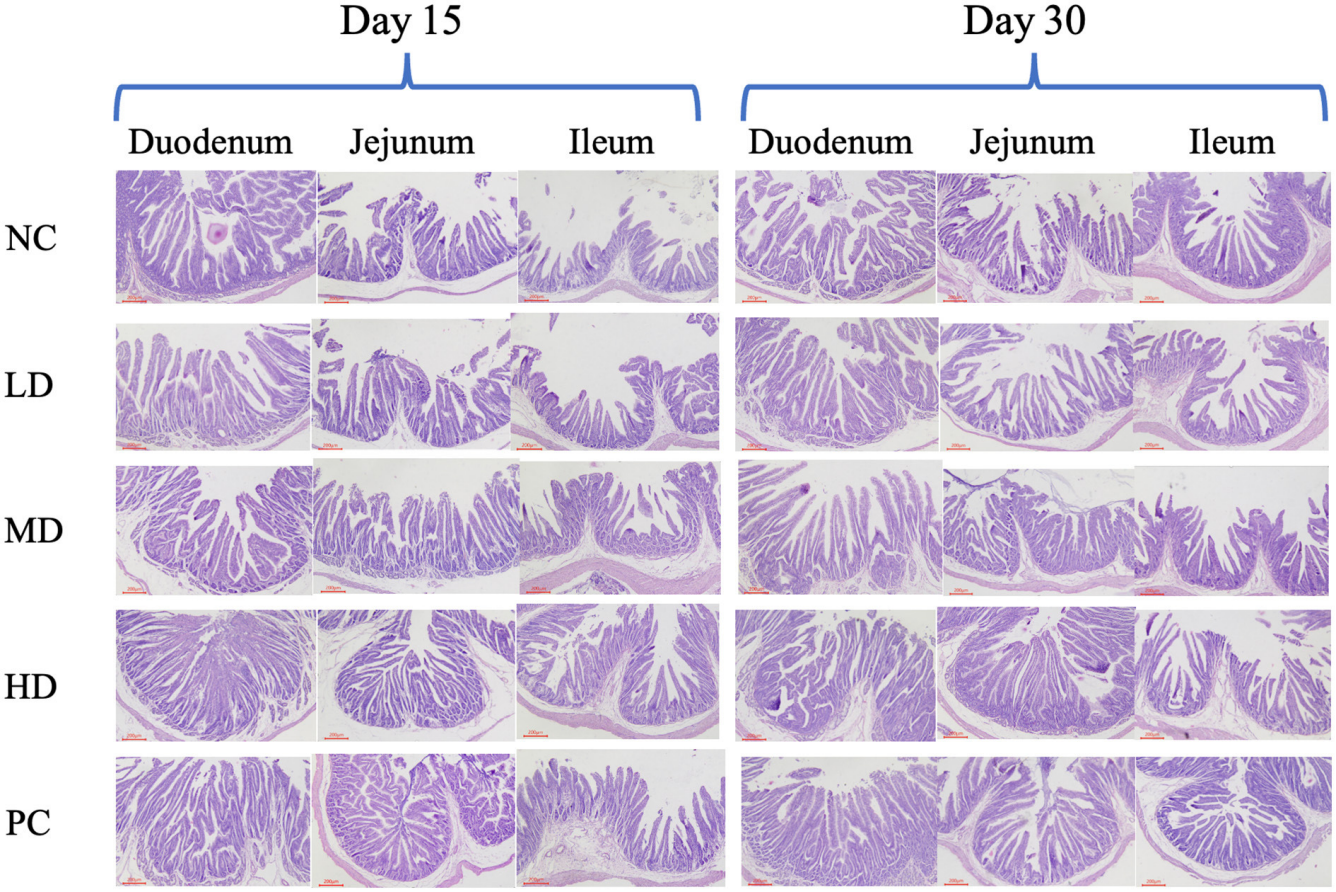
Intestinal tissue morphology was observed by H&E staining (HE × 400).

Histopathological examination was commonly used to evaluate the integrity of intestinal tissue. Collected at Day 15 and Day 30, the tissue samples of duodenum, jejunum, and ileum were made into sections for H&E staining. The 400× microscopic images are shown in Figure 3. The results of H&E staining assay demonstrated that in NC and LD groups, the part of intestinal villi shed, and the intestinal villi of intestine were shorter. The proper layer was wider, and the connective tissue of proper layer loosened. However, the results of HD and PC groups showed that the intestinal structure was integrity, stage was clear, and intestinal villus was neatly arranged in all groups. The tissue structure of the samples in MD group was normal, with no obvious histopathological damage found, and the mucosal epithelium showed cell abscission and necrosis occasionally. As shown in Tables 4–6, our studies showed that SJ-SJZG could increase the length of villi, the depth of crypt, and their radio, which is mentioned as R_(V/C)_, and there was a dose-dependent effect in the LD, MD, and HD dose groups. Moreover, the villi length and R_(V/C)_ were remarkably risen in jejunum in HD group at Day 15, and R_(V/C)_ of PC group also increased significantly compared with the NC group at Day 15 (Table 5). As a whole, SJ-SJZG had better protection on intestinal mucosal mucosa at Day 15. The intestinal development of Rex rabbits in Day 15 was not sufficient compared the Rex rabbits at Day 30. The young rabbits could fully reflect the development and protection of SJ-SJZG on intestinal mucosal structure.

**Table 4:** Effects of SJ-SJZG on R_(V/C)_ in duodenum of weaned Rex rabbits.

**Time**	**Item**	**Group**				
		**NC**	**LD**	**MD**	**HD**	**PC**
Day 15	L_villi_ (μm)	648.94 ± 46.31	659.42 ± 21.11	682.06 ± 76.20	701.86 ± 40.39	698.93 ± 37.18
Day 30		722.12 ± 47.55	745.91 ± 37.83	754.38 ± 44.28	762.19 ± 38.63	772.96 ± 55.47
Day 15	D_crypt_ (μm)	100.53 ± 6.62	98.50 ± 7.68	97.39 ± 1.28	94.00 ± 8.41	96.74 ± 9.39
Day 30		92.93 ± 4.49	92.87 ± 5.14	92.52 ± 4.95	91.14 ± 5.01	90.14 ± 8.13
Day 15	R _(V/C)_	6.46 ± 0.32	6.72 ± 0.50	7.01 ± 0.87	7.48 ± 0.28	7.27 ± 0.78
Day 30		7.79 ± 0.70	8.04 ± 0.51	8.17 ± 0.60	8.39 ± 0.86	8.59 ± 0.29

Data are expressed as mean ± SD (n = 6).

**Table 5:** Effects of SJ-SJZG on R_(V/C)_ in jejunum of weaned Rex rabbits.

**Time**	**Item**	**Group**				
		**NC**	**LD**	**MD**	**HD**	**PC**
Day 15	L_villi_ (μm)	453.29 ± 27.38^a^	457.91 ± 14.00^a^	487.47 ± 14.25^ab^	499.71 ± 21.04^b^	492.58 ± 27.44^ab^
Day 30		502.08 ± 21.98	507.90 ± 28.78	511.70 ± 26.05	520.39 ± 51.60	526.21 ± 42.03
Day 15	D_crypt_ (μm)	110.79 ± 7.44	108.76 ± 7.23	108.72 ± 6.47	107.84 ± 3.04	106.77 ± 5.35
Day 30		92.46 ± 4.06	93.18 ± 2.77	89.94 ± 3.02	90.65 ± 6.49	91.51 ± 7.21
Day 15	R_(V/C)_	4.10 ± 0.25^a^	4.23 ± 0.38^ab^	4.50 ± 0.37^ab^	4.63 ± 0.07^b^	4.62 ± 0.17^b^
Day 30		5.43 ± 0.09	5.46 ± 0.46	5.69 ± 0.38	5.77 ± 0.85	5.78 ± 0.73

Data are expressed as mean ± SD (n = 6). R_(V/C)_, Ratio of villus length to crypt depth.

**Table 6:** Effects of SJ-SJZG on R_(V/C)_ in ileum of weaned Rex rabbits.

**Time**	**Item**	**Group**				
		**NC**	**LD**	**MD**	**HD**	**PC**
Day 15	L_villi_ (μm)	420.22 ± 4.64	423.36 ± 14.79	443.51 ± 29.12	452.29 ± 18.26	447.20 ± 38.64
Day 30		472.49 ± 41.22	473.00 ± 48.95	473.51 ± 44.29	483.02 ± 19.43	485.07 ± 40.62
Day 15	D_crypt_ (μm)	112.69 ± 5.67	111.40 ± 6.18	107.61 ± 5.32	107.36 ± 3.63	107.77 ± 2.80
Day 30		87.26 ± 3.85	86.92 ± 5.90	87.83 ± 9.86	85.25 ± 2.09	86.30 ± 5.91
Day 15	R_(V/C)_	3.74 ± 0.21	3.80 ± 0.08	4.12 ± 0.24	4.22 ± 0.30	4.15 ± 0.41
Day 30		5.42 ± 0.50	5.48 ± 0.89	5.44 ± 0.89	5.67 ± 0.36	5.63 ± 0.53

Data are expressed as mean ± SD (n = 6).

### Promotion effects of SJ-SJZG on intestinal mucosal immunity

**Figure 4:**
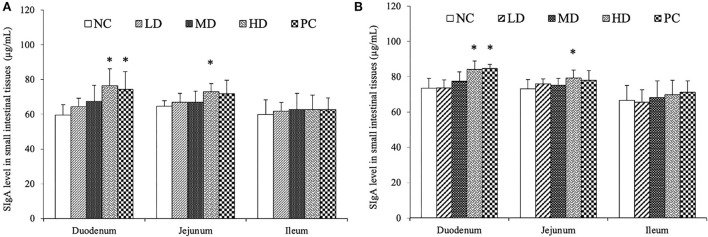
SIgA content in small intestinal tissues by Elisa, Day 15 **(A)** and Day 30 **(B)**. The data were presented as means ± S.E.M (*n* = 6). Significant difference with NC group was designated as *p < 0.05.

**Figure 5:**
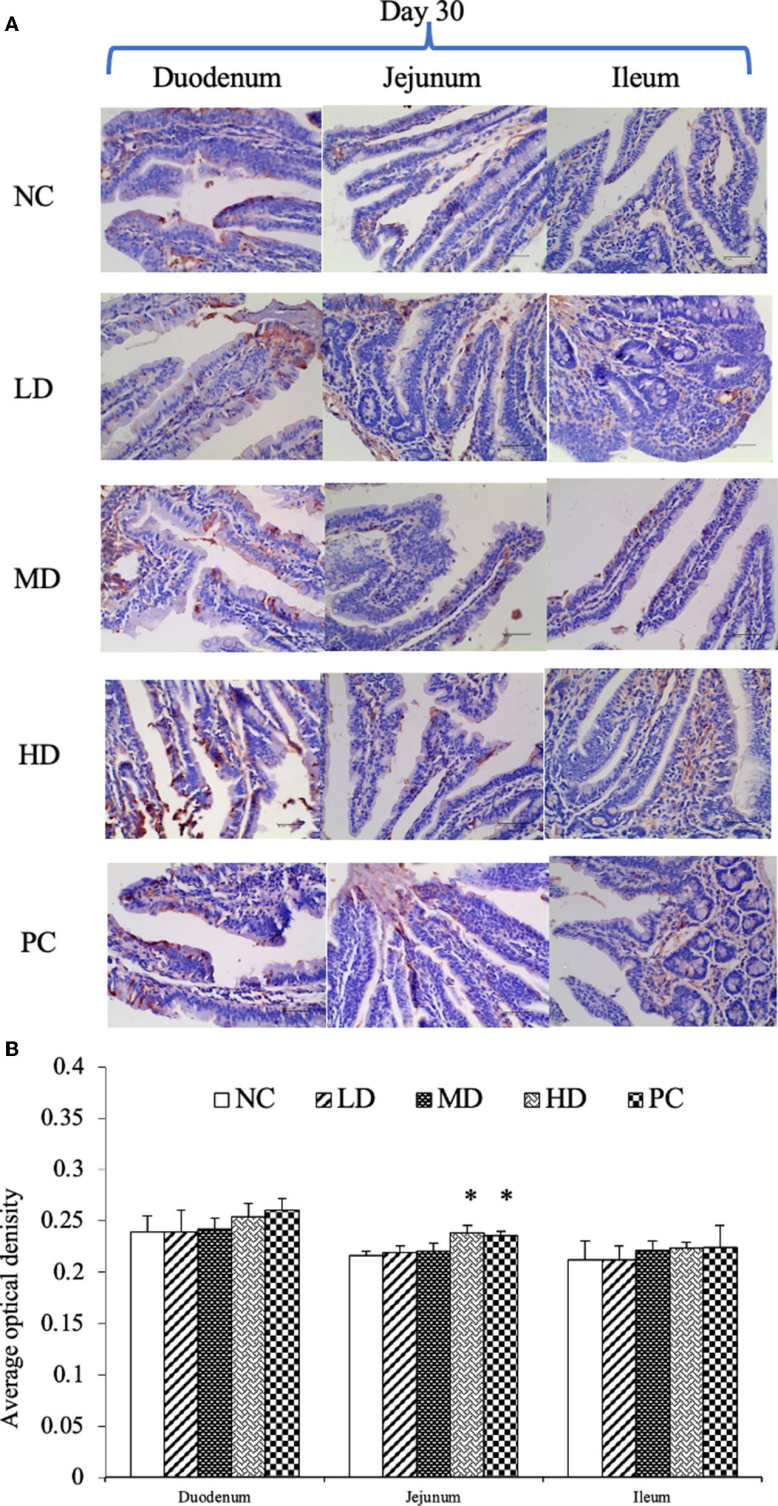
Immunohistochemical results of SIgA at Day 30 **(A)**. Average optical density of SIgA expression on small intestinal mucosa **(B)**. The data were presented as means ± S.E.M. (*n* = 6). Significant difference with NC group was designated as *p < 0.05.

SIgA, as the most important antibody in intestinal mucosal immunity, was resistant to various bacterial pathogens. We tested the content of SIgA in the small intestine of weaned Rex rabbits by ELISA. At the same time, we located and quantitatively analyzed SIgA by immunohistochemical staining. The SIgA level in intestinal mucosa was significantly increased in HD group of duodenum and jejunum at Day 15 and Day 30 (Figures 4A,B). For the PC group, the SIgA content also increased significantly in duodenum at Day 15 and Day 30 (Figures 4A,B). However, there was no significant SIgA content in ileum between all groups. In addition, SIgA level in all groups had tendency that increased with time. Immunohistochemical results showed that the SIgA positive cell was mainly distributed in the cytoplasm (Figure 5A). Immunohistochemical detection showed that SIgA protein expression of jejunum was increased significantly in HD and NC group at Day 30 (Figure 5B). The results were consistent with the results of ELISA.

### SJ-SJZG enhanced intestinal barrier function and increased the relative expression of tight junction protein, Zo1, Claudin1, and Occludin

**Figure 6:**
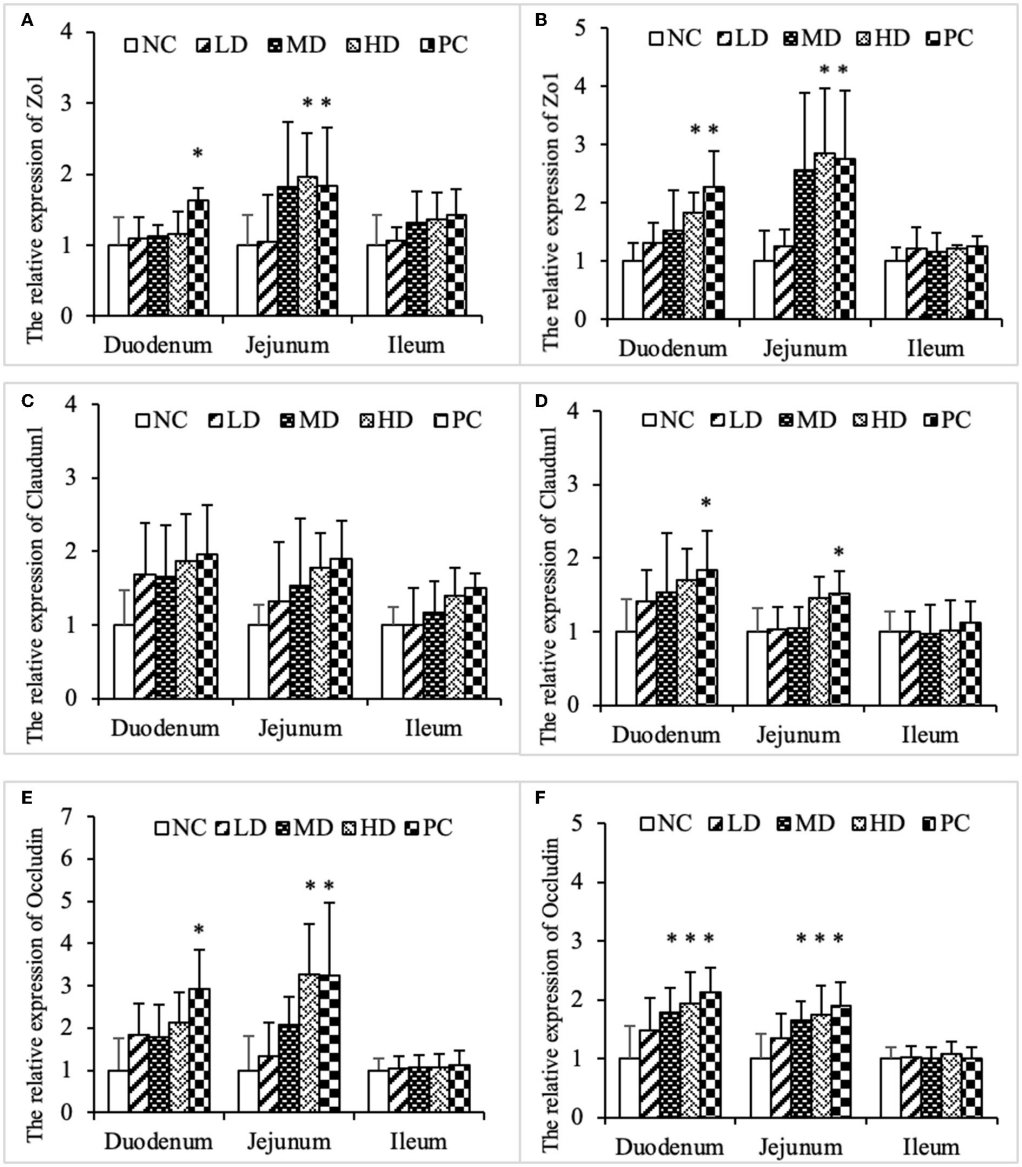
Effects ofSJ-SJZG on the relative expression of tight junction protein in small intestine mucosa. **(A)** Zo1, Day 15. **(B)** Zo1, Day 30. **(C)** Claudin1, Day 15. **(D)** Claudin1, Day 30. **(E)** Occludin, Day 15. **(F)** Occludin, Day 30. The data were presented as means ± S.E.M. (*n* = 6). Significant difference with NC group was designated as *p < 0.05.

The barrier function of intestine was closely related to tight junction protein. Zo1, claudin1, and occludin were all important members in the tight junction protein family. The expression of Zo1 in duodenum and jejunum of HD group and PC group increased significantly (Figures 6A,B). Glutamine could increase the expression of claudin1 in duodenum and jejunum (Figures 6C,D). Similarly, SJ-SJZG and glutamine significantly increased the expression of occludin in duodenum and jejunum, and the expression level increased in a dose-dependent manner (Figures 6E,F).

### SJ-SJZG enhanced the relative expression of glucose transporter, GLUT2, and SGLT1

**Figure 7:**
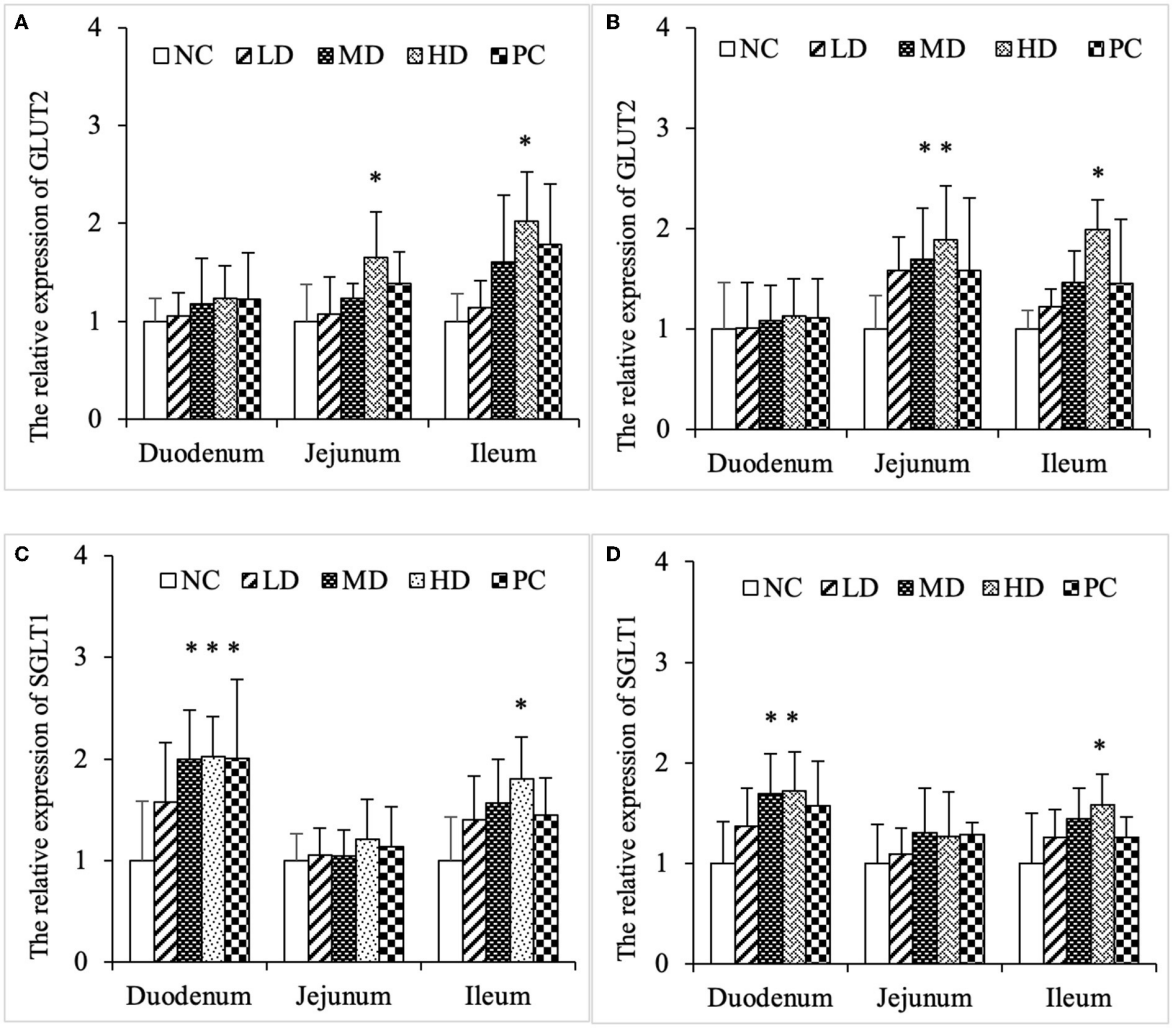
Effects of SJ-SJZG on the relative expression of glucose transporters in small intestinal mucosa. **(A)** GLUT2, Day 15. **(B)** GLUT2, Day 30. **(C)** SGLT1, Day 15. **(D)** SGLT1, Day 30. The data were presented as means ± S.E.M. (*n* = 6). Significant difference with NC group was designated as *p < 0.05.

GLUT2 and SGLT1 were two glucose transporters on intestinal mucosal epithelial cells. The results showed that SJ-SJZG could significantly increase the expression of GLUT2 in jejunum and ileum, presenting dose dependence (Figures 7A,B). Unlike GLUT2, the expression of SGLT1 caused by SJ-SJZG was mainly concentrated in duodenum and ileum. The expression of SGLT1 in duodenum of MD group and HD group was significantly higher than NC group. The relative expression of SGLT1 in ileum of high-dose group was also significantly higher (Figures 7C,D).

## Discussion

Early weanling animals often arise ablactation hyperirritability, which causes diarrhea. Antibiotic therapy, probiotic treatments, and nutrients supplement had been discussed to prevent PWD. However, PWD, as a comprehensive unhealthy state, could not be treated from a single method. Given the multifactorial and complex pathogenesis of PWD, multimodal interventions, such as the use of TCM formulas, may have potential to prevent and/or treat this poor condition. In the present study, we demonstrated the developmental effects of SJ-SJZG on small intestine in weaned Rex rabbits. Mechanistically, we found that SJ-SJZG could not only increase the villi length and R_(V/C)_ to promote the development of the small intestine, but also strengthen immune function by SIgA level boost. Moreover, epithelial cells arranged closely along the intestinal mucosal membrane owing to the expression of intestinal tight protein increased, especially in jejunum. Finally, SJ-SJZG may regulate glucose absorption by enhancing the relative expression of glucose transporter.

According to the theory of TCM, the different combinations of TCMs will be customized for treatment to various clinical symptoms. SJZD, as an ancient Chinese medicine prescription, was also modified by the addition of other TCMs. For example, network pharmacology indicated that the prescription of SJZD plus *Astragalus* was effective in treating chronic atrophic gastritis (14). Similarly, researchers added three to six other TCMs, which included *Astragalus, Citrus* × *aurantium L.*, and *Crataegus pinnatifida Bunge*, to SJZG for patients with spleen and stomach Qi deficiency syndrome (15). Jiawei SJZD, another formula containing SJZD plus *Bupleurum chinense DC, Astragalus, Citrus* × *aurantium L.*, and so on, significantly reduced the side effects of patients after chemotherapy for colon cancer and improved the immune function of patients (16). Similar to the modified SJZD above, SJ-SJZG contained *Astragalus mongholicus Bunge, Bupleurum chinense DC, Citrus* × *aurantium L.*, and *Crataegus pinnatifida Bunge (fruit)* and the formula of the original SJZD. *Astragalus mongholicus Bunge* could regulate intestinal barrier and treat Qi deficiency (17). It combination with Codonopsis pilosula can also treat colitis (18). *Bupleurum chinense DC* also had a protective effect on small intestinal injury (19). *Citrus* × *aurantium L*. and *Crataegus pinnatifida Bunge(fruit)*, as the partners of regulating Qi and strengthening stomach in TCM, had been reported to prevent intestinal diseases and protect the intestinal barrier (20, 21). The four herbs and SJZD all had protective effects on digestive tract, but there was no report of combination therapy for PWD. We proposed to add the above four TCMs into SJZD to form modified SJZD for the first time and then made it into granules for taking conveniently, which is called SJ-SJZG. The main bioactive ingredients of SJ-SJZG were saikoside A, liquiritin, hesperidin, and glycyrrhizic (Figure 2). Saikoside A, an extract from *Bupleurum chinense DC*, possessed several pharmacological activities, including anti-oxidant, anti-tumor, and protecting intestinal function (19, 22). Hesperidin, from *Citrus* × *aurantium L*., a well-known extract in TCM, had shown the various pharmacological effects of hesperidin, such as anti-inflammatory and anti-oxidation, promoting gut health and improving immunity against infections (23, 24). Liquiritin and glycyrrhizic, both from *Glycyrrhiza uralensis Fisch*, exhibited anti-inflammatory activity, which enhanced intestinal motility (25, 26). To sum up, whether from the TCM-based theory in nourishing Qi or the pharmacological activity of main components, SJ-SJZG had potential in the protection or treatment of PWD.

Our previous studies had confirmed SJ-SJZG had a beneficial effect of PWD which not only reflected in diarrhea rate and growth performance, but also the utilization of protein, carbohydrate, and lipid nutrition in body (12). However, the SJ-SJZG mechanism of reducing PWD needs to be investigated further. Therefore, we made a profound study on SJ-SJZG in preventing or protecting PWD. Moreover, glutamine was used as the positive control group. As an important conditionally indispensable amino acid, glutamine presented an important role in promoting immune function, the expression of genes related to intestinal health when the animal had been in earlier age or during stress (27). It had been confirmed that glutamine protected the intestinal health on weaned rabbits (28). Meanwhile, available evidence had suggested that dietary supplementation with glutamine improved the gene expression and immune performance on small intestinal mucosa in Rex rabbits (29). Previous studies have reported that diet supplemented with 0.8% glutamine could increase the height of small intestinal villi and reduce the depth of recess in weaned rabbits (30).

The small intestine was an important site of nutrient absorption. The longer the epithelial villi, the more the epithelial cells. The shallower the crypt depth, the higher the rate of epithelial cell maturation. The villi and crypt represented absorptive function of small intestine. Our experiment showed that both SJ-SJZG and glutamine showed the effect of increasing R_(V/C)_, especially in the jejunum (Tables 4–6). SJ-SJZG had the similar function as glutamine in protecting the integrity of small intestinal mucosa and promoting the development of small intestine. Available evidence had suggested that SIgA promoted the clearance of antigens and pathogenic microorganisms from the intestinal lumen by blocking their access to epithelial receptors, entrapping them in mucus, and facilitating their removal by peristaltic and mucociliary activities. Meanwhile, SIgA also had the capacity to directly quench bacterial virulence factors (31). In this study, we observed that SJ-SJZG significantly increased SIgA content and protein expression (Figures 4, 5). Interestingly, both the SIgA and R_(V/C)_ results showed that SJ-SJZG had the best effect on the jejunum. The protective and immunological effects of SJ-SJZG on the small intestine were mainly focused on the jejunum. SJ-SJZG could protect the integrity of jejunal mucosal epithelial cells by improving immunity in the jejunum. The intestinal barrier function referred to the sum of structure and function that can prevent harmful substances from crossing the intestinal mucosa to enter other tissue organs and blood circulation in the body. The permeability of the intestinal mucosa, as the physical barrier of the intestine, was closely related to the expression amount of tight junction proteins (32). Our study showed that SJ-SJZG increased the relative expression of tight junction protein, especially Zo1 and occludin. It was different from glutamine elevating the expression of the relative expression of Zo1, claudin1, and occludin simultaneously (Figure 6). As two glucose protein transporters with different functions, GLUT2 was responsible for passive forward concentration transport while the SGLT1 was responsible for active reverse concentration transport. According to our research, SJ-SJZG could increase the passive glucose transport capacity of jejunum and ileum by increasing the protein relative expression of GLUT2 (Figures 7A,B). SJ-SJZG can increase the active glucose transport capacity of duodenum and ileum by increasing the protein relative expression of SGLT1, but glutamine was mainly active in duodenum (Figures 7C,D). This indicated SJ-SJZG had a promoting effect on glucose absorption in the small intestine. It was conceivable that SJ-SJZG showed great potential to promote nutrient absorption in small intestine.

## Conclusion

In summary, we explored the mechanism of SJ-SJZG on small intestine developmental effects in weaned Rex rabbits. SJ-SJZG had been proved to be effective in promoting the development of small intestine and improving the immunity of small intestine. Moreover, SJ-SJZG could ensure the integrity of mucosal barrier and increase the ability of intestine to absorb glucose in small intestine. Our research provided a reliable theoretical basis for reducing the occurrence of PWD by SJ-SJZG.

## Data availability statement

The original contributions presented in the study are included in the article/supplementary material, further inquiries can be directed to the corresponding author.

## Ethics statement

The animal study was reviewed and approved by Sichuan Agricultural University Institutional Animal Care and Use Committee CSQ-2018203003.

## Author contributions

DL: methodology, data curation, and writing—original draft preparation. YW: formal analysis and validation. NL: funding support and investigation. SC and HL: visualization. PW, ZY, GS, and JL: supervision. WZ, HT, and GP: software. KZ, BW, and LZ: writing—reviewing and editing. HF: project administration. All authors read and approved the final manuscript.

## Funding

This work was financially supported by the Earmarked Fund for the China Agriculture Research System (CARS-43-A-3), Science & Technology Department of Sichuan Province (2016NYZ0046), and the basic scientific research operating expenses of Sichuan Science & Technology Department.

## Conflict of interest

The authors declare that the research was conducted in the absence of any commercial or financial relationships that could be construed as a potential conflict of interest.

## Publisher's note

All claims expressed in this article are solely those of the authors and do not necessarily represent those of their affiliated organizations, or those of the publisher, the editors and the reviewers. Any product that may be evaluated in this article, or claim that may be made by its manufacturer, is not guaranteed or endorsed by the publisher.

## Abbreviations

PWD: post-weaning diarrhea
TCM: traditional Chinese medicine
SJZD: Sijunzi Decoction
SJ-SJZD: Shugan Jianwei Sijunzi decoction
SJ-SJZG: Shugan Jianwei Sijunzi granule
NC: negative control
LD: low dose
MD: medium dose
HD: high dose
PC: positive control
HPLC: high-performance liquid chromatography
SIgA: secretory immunoglobulin A
H&E: histopathological examination
